# Sex-associated differences in baseline urinary metabolites of healthy adults

**DOI:** 10.1038/s41598-018-29592-3

**Published:** 2018-08-08

**Authors:** Sili Fan, Austin Yeon, Muhammad Shahid, Jennifer T. Anger, Karyn S. Eilber, Oliver Fiehn, Jayoung Kim

**Affiliations:** 1 West Coast Metabolomics Center, University of California, Davis, Davis, CA USA; 2 Departments of Surgery, Cedars-Sinai Medical Center, Los Angeles, CA USA; 3 Division of Urology, Department of Surgery, Cedars-Sinai Medical Center, Los Angeles, CA USA; 4 King Abdulaziz University, Jeddah, Saudi Arabia; 5 Departments of Surgery and Biomedical Sciences, Cedars-Sinai Medical Center, Los Angeles, CA USA; 6 Samuel Oschin Comprehensive Cancer Institute, Cedars-Sinai Medical Center, Los Angeles, CA USA; 7 University of California Los Angeles, Los Angeles, CA USA; 8 Department of Urology, Ga Cheon University College of Medicine, Incheon, Republic of Korea

## Abstract

The biological basis for gender variability among disease states is not well established. There have been many prior efforts attempting to identify the unique urine metabolomic profiles associated with specific diseases. However, there has been little advancement in investigating the metabolomic differences associated with gender, which underlies the misconception that risk factors and treatment regimens should be the same for both male and female patients. This present study aimed to identify biologically-meaningful baseline sex-related differences using urine samples provided by healthy female and male participants. To elucidate whether urinary metabolic signatures are globally distinct between healthy males and females, we applied metabolomics profiling of primary metabolism with comprehensive bioinformatics analyses on urine samples from 60 healthy males and females. We found that levels of α-ketoglutarate and 4-hydroxybutyric acid increased 2.3-fold and 4.41-fold in males compared to females, respectively. Furthermore, chemical similarity enrichment analysis revealed that differentially expressed metabolites, such as saturated fatty acids, TCA, and butyrates, were significantly related to the gender effect. These findings indicate that there are baseline sex-related differences in urinary metabolism, which should be considered in biomarker discovery, diagnosis, and treatment of bladder diseases, such as interstitial cystitis.

## Introduction

Interstitial cystitis (IC), also known as painful bladder syndrome (PBS), is a chronic pain disorder with no known etiology^1^. Due to the limited amount of objective diagnostic tools for IC^2,3^, there is great need to identify sensitive and non-invasive biomarkers that can vastly improve the accuracy of IC diagnoses^4^. Unfortunately, current understanding of the basic mechanisms behind pelvic pain are also fragmented^5,6^. One area of interest that may provide a wealth of information is the impact of gender on IC. Epidemiological studies have consistently demonstrated a sex-based dimorphism in IC prevalence rates^7–11^. It is generally accepted that the female to male ratio is approximately 8:2 or 9:1^7–12^. However, the reasons for this difference are currently not well understood.

One suggestion for the stark discrepancy between male and female IC prevalence rates is sexual dimorphism. Moreover, there are sex-determined differences when diseases start and develop. Although these disparities are well-noted, the biological, cellular, and molecular basis of these gender biases remain elusive. One theory is that sex hormones are possibly associated with noted variations in metabolism. This is evident in other diseases. For example, it has been reported that female cases of autoimmune disease are three to four times higher than that of males^13–15^. Another study suggested that multiple sclerosis (MS) patients showed distinct gene signatures between females and males^16^.

In addition to the aforementioned diseases, heart failure and cardiovascular disease (CVD) have been reported to be associated with sex differences^17–21^. In females, hypertension is more common and is often the cause of heart failure. However, females also have a better prognosis than males with heart failure. For CVD, blood pressure and glucose metabolism play more important roles in females, whereas male CVD is affected mostly by cholesterol. The average starting age of CVD incidence in males is around the mid-30s and gradually increases; while in females, CVD usually occurs much later, around 50 years of age. Furthermore, the plasma lipid profiles of younger-aged females are generally better than similarly aged males, which may explain why females have lower risk for CVD. In an effort to examine these sex-biases, mammalian animal models have been used to explore how males and females develop diseases differently and identify potential therapeutic targets. A study in female and male Sprague–Dawley rat models showed that hearts from female rats have better cardioprotection than male rats. Phosphorylation of mitochondrial proteins in female rats were altered, leading to less reactive oxygen species (ROS) generation and oxidative metabolism^22^.

Despite the numerous metabolic studies on various types of diseases in animal models^23,24^, gender bias in metabolic signatures has not been mechanistically investigated in the healthy human setting. One study used a metabolomics approach to identify specific urinary markers for major depressive disorder (MDD). The authors reported that male and female MDD patients showed very distinct metabonomic signatures^25^. More recently, in the cross-sectional KarMeN (Karlsruhe Metabolomics and Nutrition) study, the metabolite profile of healthy human urine was reported to be capable of predicting age and sex^26^. While research into sex differences in other diseases has progressed, the same cannot be said for lower urinary tract symptoms, such as IC or overactive bladder (OAB). The biological mechanisms underlying sex variation in various bladder dysfunctions are still not fully understood. Further investigation into the relationships among sex-specific risk factors, metabolic rewiring, IC prevalence, and symptom severity are essential for explaining these sex-related differences.

In this study, our first aim was to determine the base levels of urinary metabolites in healthy controls. We additionally attempted to test whether urinary metabolomic profiles were globally different between females and males. To achieve these goals, we performed untargeted global gas chromatography-time-of-flight-mass spectrometry (GC-TOF-MS) profiling of primary metabolism and comprehensive bioinformatics analysis. Our metabolomic profiles showed distinct patterns of differentially expressed metabolites (DEMs) and suggested an interesting list of DEMs specific to healthy females and males. Given that sex influences some of the biomarkers reported, our findings provide evidence showing that baseline gender-related differences should be considered when developing urine-based strategies for metabolomic biomarkers.

## Materials and Methods

### Ethics statement

The Ethics Committee and the Institutional Review Board of Cedars-Sinai Medical Center (CSMC) approved recruitment, sample collection, curation, and analysis of metabolomics profiling data for this study (IRB# Pro00040261). All subjects who participated in this study provided written informed consent, and all experiments were performed in accordance with relevant guidelines and regulations.

### Participants and urine collection

Healthy participants were recruited from an outpatient urology clinic at CSMC. Subjects with a history of any chronic diseases (such as any types of cancer, inflammatory conditions, diabetes, etc.) were excluded. We enrolled 60 females and 60 age-matched males in this study. All participants were >2 months “free of treatment or medication” (2015–2016).

To minimize possible contamination with vaginal or urethral cells, urine was collected using the clean catch method in a sterile environment. The de-identified samples were assigned with new identification numbers by laboratory staff in a double-blinded manner. To remove cell debris, samples were then centrifuged at 2500 rpm for 10 min.

### GC-TOF-MS analysis of urine

#### Sample pre-processing and preparation

We investigated the metabolite profiles of the individual urine samples via gas-chromatography/mass-spectrometry (GC-MS) analysis^27,28^. The Gerstel CIS4–with dual MPS Injector and Agilent 6890 GC-Pegasus III TOF MS was used for this analysis.

First, 10 µl of urine was dissolved in a 1 ml −20 °C mixture of acetonitrile, isopropanol, and water (3:3:2 v/v) at a pH of 7.0. The urine volume was adjusted between 2–10 µl to match externally measured creatinine levels based on a linear calibration curve. The solution was then vortexed at 4 °C for 5 min in 1.5 ml centrifuge tubes. Samples were additionally centrifuged for 2 min at 14,000 rcf and 500 µl of the supernatants were aliquoted. Aliquots were evaporated in a Labconco Centrivap cold trap to complete dryness. The methoximation step was done using a 10 µl solution of 40 mg/ml o-methylhydroxylamine hydrochloride (CAS:[593-56-6]; formula CH_5_NO.HCl) in pyridine (Cas:[11-0-86-1]; formula C_5_H_5_N) and shaken for 90 min at 30 °C. Then, 90 µl of a n-methyl-n-trimethylsilyltrifluoroacetamide (MSTFA) and fatty acid methyl esters (FAME) retention time markers mixture (100:1 v/v) was added and the entire solution was shaken for 30 min at 37 °C. The mixture was transferred to amber crimp auto-sampler vials with flat bottom micro-inserts. Measurements were performed on a Leco Pegasus IV TOF coupled to an Aglient 6890GC with an Agilent 6890 split/splitless injector. The column used was a Restek RTX-5Sil MS (95% dimethyl/5% diphenyl polysiloxane) with a 30 m length, 0.25 mm i.d., and 0.25 µm film thickness with a 10 m guard column. Each injection had a set volume of 0.5 µl and was done at 50 °C. The GC parameters were set to a 1 ml/min constant flow helium and an oven ramp temperature of 50 °C (1 min hold) that steadily increased to 330 °C at a rate of 20 °C/min, with a 5 min hold before cooling-down. The transfer line temperature was set to 280 °C and the spectra were recorded in the electron ionization mode at 70 eV with a source temperature of 250 °C TOF and scan range of 85–500 u.

#### Injector conditions

The Agilent 6890 GC was equipped with a Gerstel Automatic Liner Exchange System (ALEX) that included a multipurpose sample (MPS2) dual rail and a Gerstel cold injection system (CIS) (Gerstel, Muehlheim, Germany). The temperature program was set as follows: 50 °C to a final temperature of 275 °C at a rate of 12 °C/sec with a hold for 3 min. Injections were done at a speed of 10 µl/sec on a splitless injector with a purge time of 25 sec. The liner (Gerstel #011711-010-00) was changed after every 10 samples using the Maestro1 Gerstel software (version 1.1.4.18). Before and after each injection, the 10 µl injection syringe was washed 3x with 10 µl of ethyl acetate.

#### Gas chromatography conditions

A 30 m long, 0.25 mm i.d. RTX-5Sil MS column (0.25 µm, 95% dimethyl 5% diphenyl polysiloxane film) with an additional 10 m integrated guard column was used (Restek, Bellefonte, PA). A constant flow of 1 ml/min 99.99% pure helium, with a built-in purifier, was set. The oven temperature was held at 50 °C for 1 min and then ramped up to 330 °C at a rate of 20 °C/min, where it was then maintained constant for 5 min.

#### Mass spectrometer settings

A Leco Pegasus IV Time-of-Flight Mass Spectrometer was used and controlled using the Leco ChromaTOF software (vers. 4.1) (St. Joseph, MI). The transfer line temperature between the gas chromatograph and mass spectrometer was set to 280 °C. A 70 V electron impact ionization was employed with an ion source temperature of 250 °C. The acquisition rate was set to 17 spectra/sec, with a scan mass range of 85–500 Da.

#### Annotation and ID of compounds

The peak and compounds detection or deconvolution was performed with the Leco ChromaTOF software. Spectra were matched against the FiehnLib Mass Spectral and Retention Index Library^28^. Post-curation and peak replacements were performed with the in-house developed BinBase software and the sample matrix with all known and unknown compounds was exported to an excel sheet. Missing peak intensity data was automatically replaced with the raw extracted ion intensities at the target retention times for each compound, subtracted by adjacent noise levels. This way, only 2 of the 49,680 values were reported with ‘zero’ values, compound BB 109708 and creatinine. These two values were replaced with a value of 1.

#### Missing value replacement method

BinBase was used for post-curation and peak replacements and the sample matrix was exported to an excel sheet. Any missing peak intensity data was automatically replaced by the raw extract ion intensity subtracted by their adjacent noise levels. For each positively detected metabolite, the average retention time was calculated. For each chromatogram and each missing value, the intensity of the quantification ion at this retention time was extracted by seeking its maximum value in a retention time region of 1 sec and subtracting the minimum (local background) intensity in a retention time region of 5 sec around the peak maximum. The resulting report table therefore did not have any missing values^29^.

The two missing values (*ion intensity* = *0*), for compound 109708 and creatinine, were replaced with a value of 1. All peak intensities were normalized to the sum of the peak intensities of all identified metabolites to account for small errors during extraction or normalization. We found that 77% of all compounds yielded a non-normality distribution. Hence, a nonparametric univariate method, the Mann-Whitney U test, was performed to measure and discover significantly changed metabolites between the male and female urine samples. Additionally, we adopted the Benjamini-Hochberg false discovery rate (FDR) correction procedure to deal with the multiple comparison problem and ensure the reproducibility of our significant metabolites detection. Multivariate statistical analyses, principal component analysis (PCA) and partial least-square discriminant analysis (PLS-DA), were performed to discriminate males and females.

#### Statistical analysis

The Mann-Whitney U test was performed on each compound to compare males vs. females. The Benjamini-Hochberg false discovery rate (FDR) correction was utilized to deal with the multiple comparison problem.

## Results

### GC-TOF-MS analysis of urine specimens from healthy females and males

**Figure 1:**
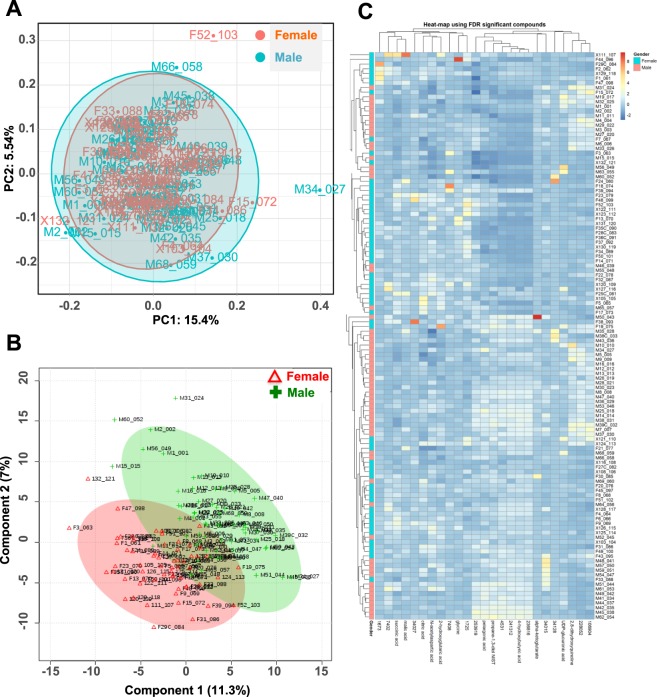
Differentiation of male and female groups using multivariate analysis. (**A**) Score plot of the PCA model distinguishing male and female urine samples. (**B**) PLS-DA scores plot. It depicted obvious differences between male and female urine samples, with PC1 (11.3%) and PC2 (7%). (**C**) Heat map showed the distribution of 25 metabolites, which include 12 annotated ones, that were significantly different (FDR adjusted p-value < 0.05) between male and female urine samples.

In total, we enrolled 60 females and 60 age-matched males (age range, 45–65) in this study. All participants were >2 months free of treatment and/or medication. In order to compare the metabolite profiles of urine samples from healthy females and males, individual urine samples were analyzed using GC-TOF mass spectrometry. Multivariate statistical analyses, PCA and PLS-DA, were performed to discriminate males and females (Fig. 1A, B).

Metabolites responsible for the differences in multivariate metabolic phenotypes between male and female urine was obtained based on variable importance in the projection (VIP) from a 10-fold cross-validated PLS-DA model. This model achieved a 48.5% rate of discrimination for Q2 and an 89.1% for R2. In addition, the model was further validated with the permutation test on prediction accuracy and had a significant result (*p* < 0.05), indicating that the model was robust and results were not obtained by chance. Significantly altered metabolites distinguishing male and female urine were acquired based on conditions of *p* < 0.05, fold-change < 0.8 or >1.2, and VIP > 1 (Fig. 1B). Hierachical clustering analysis (*Euclidean* distance and *complete* method) and constructed heatmaps, using the significant metabolites (corrected FDR *p* < 0.05), depicted the relatively disturbed and unbalanced metabolism states between male and female samples (Fig. 1C).

### Distinct urinary metabolite patterns between healthy females and males

**Figure 2:**
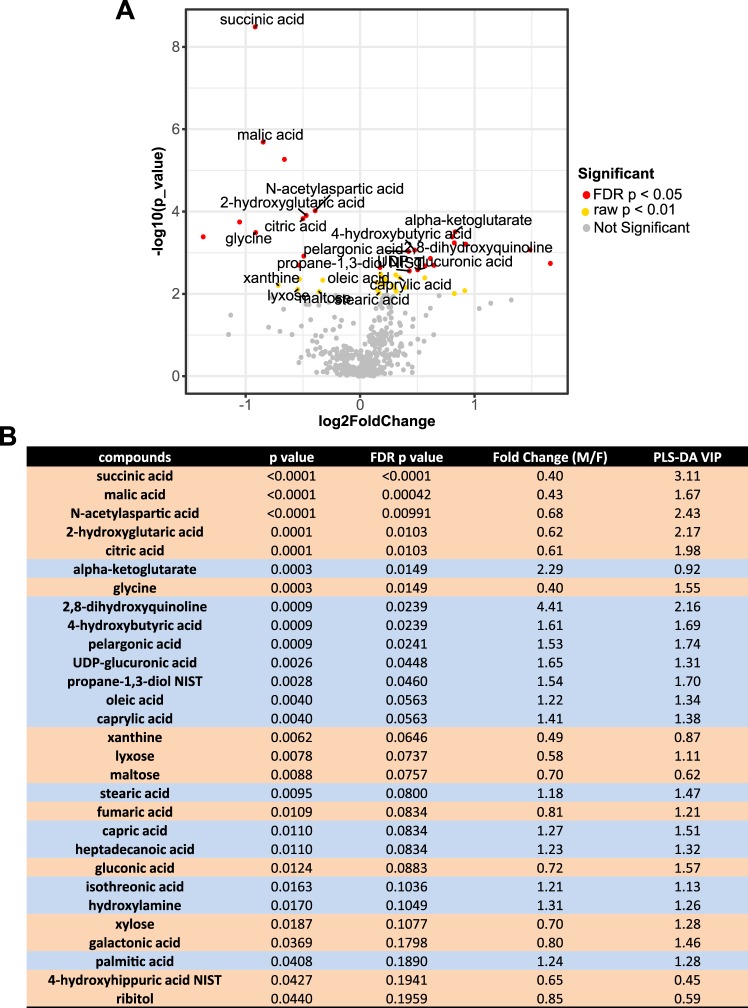
Volcano plot and significant metabolite table. (**A**) In the volcano plot, a total of 50 peaks were significantly changed (Mann-Whitney U test p-value < 0.01) in urine samples. Red dots represent 25 significant peaks with FDR adjusted p-values < 0.05. The annotated significant metabolites are labeled on the plot. (**B**) List of the 21 potential biomarkers in urine samples. P-values were calculated using two-tailed Mann-Whiney U tests. FDR p-values were p-values corrected for the multiple comparison problem using the Benjamini and Hochberg procedure. Fold changes were defined as the ratio of median of male over female for each compound. The variable importance for projection (VIP) reflects the capability of the compounds to explain Y (gender effect).

Next, we performed the Mann-Whitney U test and Benjamini-Hochberg false discovery rate (FDR) correction on each compound to compare them between males and females. There were 25 significantly different compounds with FDR correction and 94 without (Supplementary Table 1). The volcano plot shows the fold change and significance of each annotated metabolite. Significant metabolites in the volcano plot had a fold-change threshold >1.20 or <0.83 with a t-test p-value < 0.05 (Fig. 2A). The 25 significantly different compounds are shown with their p-values, FDR p-values, fold-changes of male vs. female, and PLS-DA VIP values (Fig. 2B). These compounds include succinic acid, malic acid, N-acetylaspartic acid, 2-hydroxyglutaric acid, citric acid, α-ketoglutarate and others. Among these, α-ketoglutarate (male/female fold-change of 2.29) or 4-hydroxybutyric acid (male/female fold-change 4.41) increased in healthy males compared to females. In contrast, levels of succinic acid (male/female fold-change of 0.40), malic acid (male/female fold-change of 0.43), or glycine (male/female fold-change of 0.40) greatly decreased in males compared to females.

**Figure 3:**
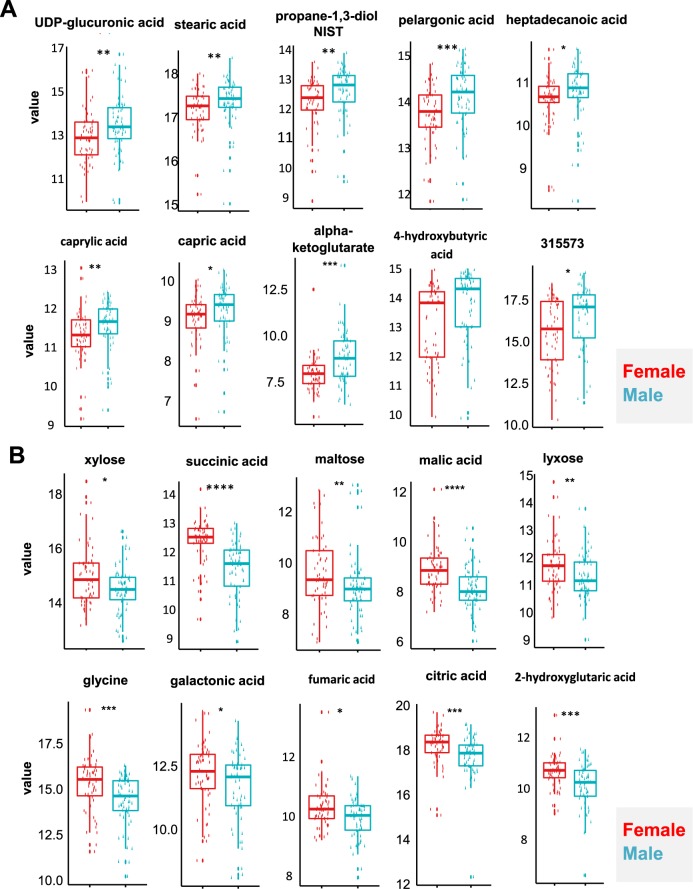
Compound individual boxplots. Boxplots showing upregulated (**A**) and downregulated (**B**) metabolites that could be used to differentiate male and female samples. Significance levels were highlighted using *(p value < 0.05), **(*p* < 0.01), ***(*p* < 0.001), and ****(*p* < 0.0001).

Significantly increased and decreased metabolites in males, compared to females, are shown in Fig. 3A and 3B, respectively. Box-whisker plots were constructed after log2 transformation. The Mann-Whitney U test was used to determine significance (*p < 0.05, **p < 0.01, or ***p < 0.005). All metabolites identified in this study are shown in Supplementary Tables 1 and 2. A few example metabolites whose expression was lower or higher in healthy females compared to healthy males are shown in Fig. 3A and 3B. Expression levels of UDP-glucuronic acid, stearic acid, propane-1,3-diol, pelargonic acid, heptadecanoic acid, caprylic acid, capric acid, α-ketoglutarate, hydroxybutyric acid, and unknown compound BB 315573 were all detected to be considerably lower in urine obtained from healthy females compared to males (Fig. 3A). In contrast, levels of xylose, succinic acid, maltose, malic acid, lyxose, glycine, galactonic acid, fumaric acid, citric acid, and 2-hydroxyglutaric acid were found to be greater in females than in males (Fig. 3B). Table 1 shows a list of metabolites differentially expressed in IC, compared to controls (p-level = 0.005, FDR (Benjamini Hochberg). A list of metabolites with PLS-DA VIP score > 1, Mann-Whitney U test p value < 0.05, and fold change (male/female) <0.8 or >1.2 are shown in Table 2.

**Table 1:** List of metabolites differentially expressed in IC, compared to controls (p-value = 0.005, FDR (Benjamini Hochberg).

Num	Name	Fold-change	p-value	FDR
1	Unknown BB_31554	2.56	0.000132	0.064576
2	Unknown BB_34163	0.55	0.000514	0.12586
3	oleic acid	0.63	0.001933	0.315675
4	2-deoxytetronic acid	1.26	0.008732	0.571396
5	Unknown BB_17651	0.66	0.009136	0.571396
6	saccharic acid	0.80	0.012642	0.571396
7	Unknown BB_17140	1.44	0.015588	0.571396
8	phosphate	0.70	0.016252	0.571396
9	trehalose	1.79	0.017026	0.571396
10	Unknown BB_5900	0.81	0.017487	0.571396
11	erythronic acid	2.25	0.018393	0.571396
12	Unknown BB_109809	0.56	0.018576	0.571396
13	oxalic acid	0.48	0.018665	0.571396
14	Unknown BB_34027	0.44	0.019904	0.571396
15	Unknown BB_1704	0.63	0.020865	0.571396
16	sulfuric acid	0.31	0.021197	0.571396
17	Unknown BB_23635	0.69	0.02138	0.571396
18	cystine	1.47	0.021607	0.571396
19	Unknown BB_3029	0.70	0.022156	0.571396
20	Unknown BB_12330	2.19	0.02596	0.614149
21	Unknown BB_31549	0.37	0.026702	0.614149
22	lyxitol	1.42	0.028288	0.614149
23	Unknown BB_31756	1.74	0.028827	0.614149
24	lysine	1.49	0.034624	0.706901
25	histidine	1.79	0.040576	0.743323
26	Unknown BB_31359	0.81	0.043988	0.743323
27	Unknown BB_5121	0.64	0.045462	0.743323
28	Unknown BB_100869	1.58	0.046685	0.743323
29	Unknown BB_3294	1.37	0.046907	0.743323
30	Unknown BB_31764	1.33	0.048566	0.743323

**Table 2:** List of metabolites with a PLS-DA VIP score > 1, Mann-Whitney U test p-value < 0.05, fold-change (male/female) <0.8 or >1.2.

label	PubChem	PLS-DA VIP Score	p_value	p_value_adj	Fold Change
xylose	135191	1.28	0.0187	0.1077	0.70
UDP-glucuronic acid	17473	1.31	0.0026	0.0448	1.65
succinic acid	1110	3.11	0.0000	0.0000	0.40
propane-1,3-diol NIST	10442	1.70	0.0028	0.0460	1.54
pelargonic acid	8158	1.74	0.0009	0.0241	1.53
palmitic acid	985	1.28	0.0408	0.1890	1.24
oleic acid	445639	1.34	0.0040	0.0563	1.22
N-acetylaspartic acid	65065	2.43	0.0001	0.0099	0.68
malic acid	525	1.67	0.0000	0.0004	0.43
lyxose	439240	1.11	0.0078	0.0737	0.58
isothreonic acid	151152	1.13	0.0163	0.1036	1.21
hydroxylamine	787	1.26	0.0170	0.1049	1.31
heptadecanoic acid	10465	1.32	0.0110	0.0834	1.23
glycine	750	1.55	0.0003	0.0149	0.40
gluconic acid	6857417	1.57	0.0124	0.0883	0.72
galactonic acid	128869	1.46	0.0369	0.1798	0.80
citric acid	311	1.98	0.0001	0.0103	0.61
caprylic acid	379	1.38	0.0040	0.0563	1.41
capric acid	2969	1.51	0.0110	0.0834	1.27
4-hydroxybutyric acid	10413	1.69	0.0009	0.0239	1.61
2-hydroxyglutaric acid	43	2.17	0.0001	0.0103	0.62
2,8-dihydroxyquinoline	97250	2.16	0.0009	0.0239	4.41

We speculated that metabolite enrichment analysis may be able to provide the corresponding gender-specific pathways derived from the specific metabolites on their differential networks. We performed metabolite set enrichment analysis (MSEA) with the 29 significant metabolites (Mann-Whitney U test p-value < 0.05). We found that differentially expressed metabolites in female and male groups were highly related to α-ketoglutarate dehydrogenase complex deficiency, with a FDR of 0.00524 (raw p, 0.0000181; Holm p, 0.00524) (Supplementary Fig. 1).

**Figure 4:**
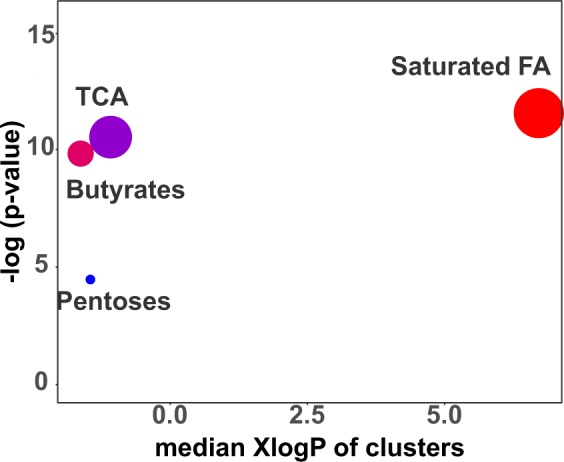
Chemical similarity enrichment analysis results. Y-axis shows the most significantly altered clusters on the top. Cluster color gives the proportion of increased or decreased metabolites (red = increased, blue = decreased, purple = mostly decreased). Chemical enrichment statistics was calculated using Kolmogorov–Smirnov test. Only significantly different enrichment clusters (raw *p* < 0.05) were shown.

Recently, we published a paper demonstrating that the hypergeometric test could be flawed for metabolomics analyses and should not be used^30^. We also noted that MSEA may be faulty as well. Thus, in this study, we opted to utilize the newly developed chemical similarity enrichment analysis (ChemRICH)^30^ instead (http://chemrich.us). ChemRICH is a chemical similarity-based statistical enrichment tool with better subsequent enrichment statistics than pathway enrichments and is not dependent on biochemical knowledge annotations. However, it does not provide information regarding enzymes or diseases. To better understand the metabolic signature specific to female and male groups, we performed ChemRICH using the Mann-Whitney U test p-values and median fold-changes of our 140 identified metabolites. ChemRICH was implemented using the Kolmogorov–Smirnov test on the identified clusters to evaluate whether a metabolite cluster was represented more than expected by chance. As a result, we found that saturated fatty acids (FA) (raw *p*, 0.0000096; FDR *p*, 0.00018), TCA (raw *p*, 0.000027; FDR *p*, 0. 0.00025), and butyrates (raw *p*, 0.000054; FDR *p*, 0.00034) were significantly related (FDR *p* < 0. 05) to the gender effect (Fig. 4, Table 3).

**Table 3:** Chemical similarity enrichment analysis.

Cluster name	Cluster size	p-values	FDR	Increased	Decreased	Increased ratio	Altered Ratio
Saturated FA	10	0.0000096	0.00018	6	0	0.6	0.6
TCA	8	0.000027	0.00025	1	5	0.1	0.8
Butyrates	5	0.000054	0.00034	2	0	0.4	0.4
Pentoses	4	0.011	0.054	0	2	0	0.5
Sugar Acids	9	0.26	0.99	0	2	0	0.2
Amino Acids	7	1	1	0	1	0	0.1
Benzoates	3	1	1	0	0	0	0
Cinnamates	3	1	1	0	0	0	0
Dicarboxylic Acids	3	1	1	0	0	0	0
Disaccharides	4	1	1	0	1	0	0.2
Gum Arabic	3	1	1	0	0	0	0
Hexoses	7	1	1	0	0	0	0
Hippurates	3	1	1	0	1	0	0.3
Indoles	4	1	1	0	0	0	0
Phenols	5	1	1	0	0	0	0
Phenylacetates	4	1	1	0	0	0	0
Purinones	3	1	1	0	1	0	0.3
Pyrimidinones	3	1	1	0	0	0	0
Sugar Alcohols	14	1	1	0	1	0	0.07

Cluster name is redefined metabolite chemical clusters. Cluster size indicates the size of the cluster. P-values are the result of the Kolmogorov–Smirnov test evaluating how significant difference a metabolite cluster was represented by chance. FDR is the Benjamini-Hochberg corrected p values. The Increase/ratio (Decreased/ratio) shows the numbers/ratio of directions of significant compounds in a cluster.

## Discussion

Our objective in this study was to systematically examine sex differences in urine samples obtained from healthy participants. The experimental results from this study provided evidence suggesting that gender influences the global metabolome in healthy female and male subjects. It has been generally accepted that pathogenesis in females may be influenced by metabolic perturbations caused by various hormones, such as estrogen, and other reproductive factors. Although further research is needed, levels of sex hormones may be related to the differences in metabolites detected in the urine of healthy adults.

As proposed in our previously published review articles, monitoring the changes in metabolic landscape, cholesterol, and sex hormones through metabolic profiling can be of immense benefit to patients^31,32^. Our urinary metabolomics data clearly suggests that sex differences should be considered in future laboratory and clinical studies. Sex has not been heavily considered as an important factor when it comes to identifying druggable targets in diseases, resulting in few biomarker studies that actively look at sex. Since gender is an influencing factor on pharmacological responses, which in turn is monitored through biomarker changes, careful should be done when consideration should be given when developing effective treatments for males and females.

Levels of urinary metabolites, including UDP-glucuronic acid, α-ketoglutarate, and 2-hydroxyglutaric acid, were found to be higher or lower in females than in males (Fig. 3). We further hypothesized the biology associated with sex-specific metabolites detected in urine samples is linked with sex hormones. Our data suggested the level of UDP-glucuronic acid is higher in healthy males, compared to females. UDP-glucuronic acid is oxidized from UDP-glucose and NAD^+^ by UDP-glucose dehydrogenase (UGDH). Considering that the expression of UGDH is known to be stimulated by androgen, a male sex hormone^33^, it would be interesting to see if androgen causes the differential levels of UDP-glucuronic acid found in urine samples of female vs. males. In addition, 2-hydroxyglutaric acid (2HG) was found to be greater in females than in males (Fig. 3B). 2HG is known as an oncometabolite that can accumulate in estrogen receptor-negative breast tumors^34^, suggesting a potential association between increased 2HG levels and estrogen, a female sex hormone.

Previous work from our laboratory and others proposed a series of urinary metabolites as potential IC biomarker candidates^35–40^. Compared to healthy controls, these metabolites significantly increased or decreased in IC patient urine samples^36^. One metabolite of interest that we found was α-ketoglutarate, which is an important TCA cycle product and is involved in lipid and acetate metabolism. It is also an epigenetic regulator, controlling transcription and translation of DNA through histone acetylation. Treatment with α-ketoglutarate slowed cell proliferation in normal bladder epithelial cells^39^. This is consistent with prior clinical observations suggesting that there are thinner layers of bladder epithelium in IC^41,42^. It was interesting that our current study found higher levels of α-ketoglutarate in healthy males, compared to females. Furthermore, a recent finding from our laboratory suggested the molecular mechanism through which α-ketoglutarate epigenetically regulates bladder epithelial cells^43^.

Unfortunately, in this current study, we could not provide solid experimental evidence explaining why healthy male urine samples contained these higher levels of α-ketoglutarate. Furthermore, considering that previous results from our group were based only on female participants, expansion of our earlier findings in both sexes would be warranted in the near future.

In summary, our findings suggested that baseline sex-determined differences would be helpful in identifying sex specific biomarkers. Currently, gender differences have not been carefully considered as important confounding factors for biomarker development. Our results provide evidence demonstrating otherwise; drug development and therapies may need more precise and detailed experimental designs that recognize the effects of sex differences on therapeutic efficacy.

## Electronic supplementary material

Supplementary Figure 1

Supplementary Table 1

Supplementary Table 2
