# Supplementary material for: Sex-associated differences in baseline urinary metabolites of healthy adults
Source: Sci Rep. 2018 Aug 8;8:11883. doi: 10.1038/s41598-018-29592-3 (PMC6082868; doi:10.1038/s41598-018-29592-3)
Supplement: Supplementary file 1 — Supplementary Figure 1 [file 41598_2018_29592_MOESM1_ESM.pdf]

Sex-associated differences in baseline urinary metabolites of healthy adults

Sili Fan[1], Austin Yeon[2], Muhammad Shahid[2], Jennifer T. Anger[3], Karyn S. Eilber[3], Oliver Fiehn[1,4], Jayoung Kim[2,5,6,7,8,¶]

[1]West Coast Metabolomics Center, University of California, Davis, CA, USA; [2]Departments of Surgery, Cedars-Sinai Medical Center, Los Angeles, CA; [3]Division of Urology, Department of Surgery, Cedars-Sinai Medical Center, Los Angeles, CA, USA; [4]King Abdulaziz University, Jeddah, Saudi Arabia; [5]Departments of Surgery and Biomedical Sciences, Cedars-Sinai Medical Center, Los Angeles, CA; [6]Samuel Oschin Comprehensive Cancer Institute, Cedars-Sinai Medical Center, Los Angeles, CA, USA; [7]University of California Los Angeles, CA, USA; [8]Department of Urology, Ga Cheon University College of Medicine, Incheon, Republic of Korea

[¶]Correspondence:
Jayoung Kim, PhD.
Departments of Surgery and Biomedical Sciences, Cedars-Sinai Medical Center, 8700 Beverly Blvd., Los Angeles, CA 90048
Tel: +1-310-423-7168   Fax: +1-310-967-3809
E-mail: Jayoung.Kim@cshs.org

Key words:
urinary biomarkers; mass spectrometry; metabolomics; healthy adults, interstitial cystitis

SUPPLEMENTARY LEGENDS

Supplementary Figure 1. Metabolite Set Enrichment Analysis (MSEA) Summary plot (A) and table (B) for the MSEA are ranked by Holm p-value. The horizontal bars summarize the main metabolite sets identified in this analysis; the bars are colored based on their p-values and the length is based on the -fold enrichment.

A

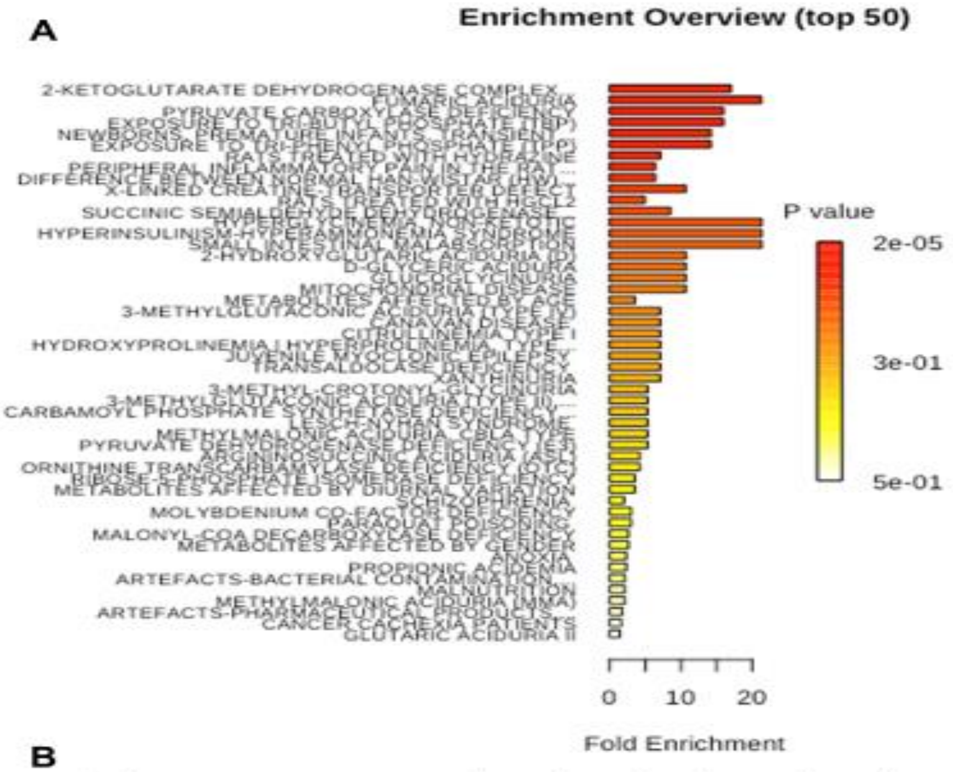

B

		total	expected	hits	Raw p	Holm p	FDR
1	2-KETOGLUTARATE DEHYDROGENASE COMPLEX DEFICIENCY	5	0.235	4	1.81E-05	0.00524	0.00524
2	FUMARIC ACIDURIA	3	0.141	3	9.09E-05	0.0263	0.0132
3	PYRUVATE CARBOXYLASE DEFICIENCY	4	0.188	3	0.000352	0.101	0.0255
4	EXPOSURE TO TRI-BUTYL PHOSPHATE (TBP)	4	0.188	3	0.000352	0.101	0.0255
5	NEWBORNS, PREMATURE INFANTS, TRANSIENT IMMATURITY OF TRANSPORT SYSTEMS OR ENZYMES	3	0.141	2	0.00615	1	0.262

Supplementary Table 1. Metabolomics data

Supplementary Table 2. Quality control data. **We calculated the relative standard deviation of these QC samples. The median of RSD of QC was 24.5%.**
